# Cross-Modal Distortion of Time Perception: Demerging the Effects of Observed and Performed Motion

**DOI:** 10.1371/journal.pone.0038092

**Published:** 2012-06-12

**Authors:** Joachim Hass, Stefan Blaschke, J. Michael Herrmann

**Affiliations:** 1 Research Group Computational Neuroscience, Central Institute of Mental Health, Medical Faculty Mannheim/Heidelberg University, and Bernstein Center for Computational Neuroscience Heidelberg-Mannheim, Mannheim, Germany; 2 FH Arnstadt-Balingen, Balingen, Germany; 3 Institute of Perception, Action and Behaviour, School of Informatics, University of Edinburgh, Edinburgh, United Kingdom; 4 Bernstein Center Göttingen, Göttingen, Germany; Duke University, United States of America

## Abstract

Temporal information is often contained in multi-sensory stimuli, but it is currently unknown how the brain combines e.g. visual and auditory cues into a coherent percept of time. The existing studies of cross-modal time perception mainly support the “modality appropriateness hypothesis”, i.e. the domination of auditory temporal cues over visual ones because of the higher precision of audition for time perception. However, these studies suffer from methodical problems and conflicting results. We introduce a novel experimental paradigm to examine cross-modal time perception by combining an auditory time perception task with a visually guided motor task, requiring participants to follow an elliptic movement on a screen with a robotic manipulandum. We find that subjective duration is distorted according to the speed of visually observed movement: The faster the visual motion, the longer the perceived duration. In contrast, the actual execution of the arm movement does not contribute to this effect, but impairs discrimination performance by dual-task interference. We also show that additional training of the motor task attenuates the interference, but does not affect the distortion of subjective duration. The study demonstrates direct influence of visual motion on auditory temporal representations, which is independent of attentional modulation. At the same time, it provides causal support for the notion that time perception and continuous motor timing rely on separate mechanisms, a proposal that was formerly supported by correlational evidence only. The results constitute a counterexample to the modality appropriateness hypothesis and are best explained by Bayesian integration of modality-specific temporal information into a centralized “temporal hub”.

## Introduction

Time is a perceptual quantity that abstracts from sensory modality - humans can judge durations of visual, auditory and multi-sensory stimuli. Yet, it is unclear how temporal information from different cues is combined to form an integrated percept of time. While a large body of literature exists on the multi-sensory representations of spatial stimulus features, such as position or size [1], only few studies examine such interactions in the temporal domain. The existing studies mostly focus on single points in time, called events, and study the perceived order or simultaneity of two such events which are marked by multi-sensory cues [2], [3]. This can be compared to the spatial notion of position - where in time does a specific event happen, relative to other events?

Duration, on the other hand, the temporal equivalent to size or length, has received much less attention in the context of multi-sensory integration, although estimates of duration are crucial for everyday life. Often, perceptional cues from different modalities carry information about the duration of an event, e.g. in speech comprehension, where we use both heard speech and lip movements as cues. To date, mainly two studies have explicitly examined cross-modal interaction of duration cues [4], [5]. Some others also considered such interactions in sequences with changing temporal frequencies [6]–[9], and the tasks in these studies may be considered as multiple duration judgments [10].

Cross-modal interactions can be examined by manipulating a stimulus presented in one modality and observing the influence of this manipulation in another one. This is either done by presenting stimuli of conflicting durations in different modalities [6]–[9], or by manipulating non-temporal stimulus features in one of the modalities [4], [5]. The latter approach is based on the fact that subjective duration is influenced by non-temporal factors, such as size or intensity of the stimulus, and by factors of attention [11]–[15]. Specifically, moving visual stimuli have been shown to appear as longer in duration compared to static ones, and this effect increases as the motion gets faster [12], [14], [16]. As an explanation, researchers have proposed that the representation of time relies on the density of events occurring during an interval [11], [12], [14], [15]. In this view, events such as the change of a stimulus are the basic units of perceived time, and the subjective duration emerges by counting these events (cf. [13], [17], [18]). Thus, the more events take place in a given period of time, the longer this period appears. Such distortions of subjective duration by non-temporal factors can be exploited to probe cross-modal interaction: If a manipulation of a stimulus in one modality results in the distortion of subjective duration in another modality, this provides evidence for a link in time perception between those modalities.

The existing studies on cross-modal representations of duration suffer from a number of severe problems. First of all, their results do not necessarily imply a direct interaction between the modalities. Because all of them explicitly presented intervals of time in each of the tested modalities, it may be that temporal representations in the more precise modality could simply override the one in the inferior modality in some cases, leaving the apparent modulation as a statistical artifact. Also, the current approaches to assess cross-modal interactions may be confounded by attentional factors. Attended stimuli are perceived as longer than unattended ones [19], [20], so the observed distortions may be due to increased saliency induced by changes in the non-temporal stimulus features, or to distraction because of the inter-modal conflict in duration, rather than direct interaction between modalities. And finally, the results of the existing studies are inconsistent. The majority of studies [2], [5], [6], [8], [9] show that the subjective duration of visually presented intervals is influenced by temporal stimuli in the auditory domain, but not vice versa. This has be taken as support of the “modality appropriateness hypothesis” [21], stating that auditory perception is dominant over visual perception in the time domain because the auditory system is superior to the visual one in terms of temporal precision. However, other studies find exactly the opposite pattern [4], or even both [7]. These potential confounds and conflicting results currently defy a straightforward account for cross-modal interactions in time perception.

The present study investigates cross-modal time perception in the sub-second range within a novel experimental paradigm. We combined a visually guided motor task with a concurrently performed auditory time perception task. Participants were required to perform an arm movement with a robotic manipulandum to follow an elliptic trajectory prescribed by a moving target on a screen. During motion, two auditory stimuli were presented which should be discriminated according to their duration. If visual and auditory temporal representations interact, the speed of visually observed motion should influence the subjective duration of auditory stimuli [12], [14], [16]. On the other hand, the visual domain does not contain any information about the onset and offset of the intervals to be timed, so in order to produce a duration distortion, the two modalities truly have to interact. The fact that participants perform a motor task also opens two additional paths of investigation. First, the tracking motion constitutes a secondary task which may interfere with time perception and impair discrimination performance [19], [20]. Thus, by manipulating the cognitive load of this task, one can test whether attentional factors play a role in the duration distortion.

Second, the tracking task allows us to assess another possible temporal cue that has been mostly neglected so far - the perception of self-motion. As the brain is able to predict and control the dynamics of the body with very precise timing [22], self-motion may also be used to estimate durations. If such a connection exists, motion parameters such as speed of curvature should extend an influence on subjective duration: Changes in motion speed could influence the density of events as in visual motion [14], while motion of higher curvature is believed to require a higher density of motor control events in the brain [23]. Previous studies which link time perception and motor timing mainly investigated whether a common timing mechanism could underly both modes of temporal processing [24]–[27], but the inverse question of whether self-motion may influence time perception has not been asked. Moreover, the current evidence for a connection between those two domains is restricted to the analysis of correlations in measures of performance for time perception and motor timing tasks [24]–[27]. As correlations can potentially be generated or concealed by other factors, a distortion of subjective duration which is caused by a motor task would provide much more solid evidence for such a connection. Thus, our paradigm allows to assess both cross-modal time perception and the possible connection between motor timing and time perception within the same sample of participants.

## Methods

### Participants

20 adult volunteers took part in each of the experiments 1 to 4, while control Experiment 1b comprised 10 participants (90 participants in total, 76 women and 14 men, mean age 23.3 years, ranging from 19 to 40 years). All had normal or corrected-to-normal vision and normal hearing. They were naïve to the purpose of the experiment. The experiment was approved by the ethics committee of the Georg-Elias-Müller Institute for Psychology of the University of Göttingen. Specifically, as there were no harmful, deceptive or otherwise ethically problematic aspects to the experiment, informed consent was received orally from each participant, documented by a list containing the names of the participants. This procedure was explicitely approved by the ethics committee.

### Apparatus and Stimuli

All experiments were controlled by a C/C++ program running on a computer operating on SuSE Linux 9.0 (SuSE Linux) and a haptic device (Phantom Premium 3.0L 6DOF, SensAble Technologies). The internal clock of the haptic device, which updates the recorded state of the robotic arm with a frequency of 1 kHz, controlled the timing of the experiments.

Participants performed all experiments standing 50 cm away from of a computer screen (Fujitsu-Siemens Computers, Scenicview P19-2), with the end effector of the robotic arm in the right hand, and wearing headphones (Technics RP-FT30). In experiments containing a motion task, arm motion was performed in the frontal plane, and recorded in all three dimensions by the haptic device (see Figure S1). In experiments containing a time perception task, participants listened to white-noise bursts generated with an external sound generator and presented binaurally through the headphones with an intensity of 65 dB(A). To avoid interference of the arm movements with a motor response, the participants responded verbally to the time perception task and their responses were recorded by the experimenter.

In all experiments, participants were presented with a setup on the screen (see Figure S2) containing a blue sphere (called “target”) and a red sphere (the “proxy”), both at 0.8 cm width and height in screen coordinates, and two ellipses drawn in yellow (main axes 22 and 12 cm for the larger ellipse, 18 and 8 cm for the smaller one). While the proxy could be controlled by moving the end effector of the robotic arm (see Figure S1), the target sphere moved clockwise on an elliptic trajectory (main axes 20 and 10 cm) that was surrounded by the yellow ellipses. In all experiments except Experiment 2, the angular velocity of the sphere was constant at 2 rad/sec. This results in a tangential velocity that varies periodically between 20 cm/sec at the upper and lower apices and 10 cm/sec at the left and right apices. In Experiment 2, the tangential velocity was kept constant to 15 cm/sec. The total time for a full revolution was 3.14 sec in all experiments.

### General Procedure

In each of the experiments, participants performed three different conditions termed “Time”, “Motion” and “Time-Motion”. Before the main experiment, they passed a short training phase on both the Time and the Motion task. The order of the Motion and the Time-Motion condition was counterbalanced among participants, while Time was always the second condition. The total experiment took about one hour. Breaks of one minute duration were taken every five minutes, or earlier if the participants requested it. After the experiment, participants were debriefed and given opportunity to ask further questions.

In the Motion condition, the participants had to track the target sphere with the proxy by moving the end of the robot arm with their hand in the plane of the screen (see Figure S1). Because of the elliptic form, the trajectory of the target motion was more curved in the left and right apex (called “curves” in the following), compared to the upper and lower one (called “straights”, see Figure S2). Participants were instructed to follow the target as closely as possible, but also to maintain a smooth, elliptic movement. Measurements confirmed that participants largely confined their motion to a plane. In the Motion task, we recorded the motion of the participants for five minutes. From the recorded trajectories, we computed the curvature and tangential velocity of the motion at each point in time (see Supporting Text S1 for the details of the computation).

The Time condition contained a duration discrimination task. This paradigm is better suited for short intervals below one second as compared e.g. to temporal reproduction [28], and less prone to motor variability. In each trial, participants compared the duration of two intervals filled with auditory noise, telling the experimenter which of the intervals appeared to be longer. The first interval *S*_1_ was always 100 ms long and the second one, *S*_2_, varied in duration according to an adaptive staircase procedure [29] to estimate the durations *S*_25_ and *S*_75_ at which the probability of judging the first stimulus as longer was .25 and .75, respectively. From these values, we computed the difference limen 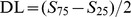 and the point of subjective equality 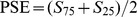. The Time condition comprised 64 pairs of stimuli in total. No feedback about the discrimination performance was given to the participants. To make the Time condition comparable to the Time-Motion condition, the elliptic motion of the target used in the Motion condition was also visible in the Time condition, and the intervals were presented at specific parts of the motion, namely the four apices of the ellipse (see Figure S2). The apex for *S*_1_ was chosen at random and *S*_2_ was presented at the apex which directly followed the one of *S*_1_. Unlike the Time-Motion condition (see below), the spatial position of the target was ignored for the variation and analysis of the intervals.

Finally, in the Time-Motion condition, participants performed the time and the motor task simultaneously, which makes Time-Motion a dual task condition compared to the two preceding single task conditions. Participants were instructed to follow the elliptic motion and to judge the duration of the intervals, both with same priority. The intervals were again presented at the four apex positions of the ellipse, but now *S*_2_ was varied independently for the four different configurations of the stimuli, which are defined by the position of *S*_2_ appearing at each of the four apices, and are named correspondingly upper and lower Straight and left and right Curve. The four conditions were presented in randomized order. In the following, we average all measures of performance over the two straights and curves conditions, which effectively leave us with the two conditions which differ in motion parameters, and are called “Straights” and “Curves”. The validity of the averaging was confirmed in a control experiment, described in the Supporting Text S1 (Experiment 1b). For each experiment, we also report the PSE and DL values for the individual apices in the Tables S1 and S3. We used 64 trials to estimate *S*_25_ and *S*_75_ for each of the four conditions, resulting in a total of 256 trials.

Specific changes of the procedure in each of the individual experiments are described in the corresponding parts of the results section.

## Results

### Experiment 1

**Figure 1:**
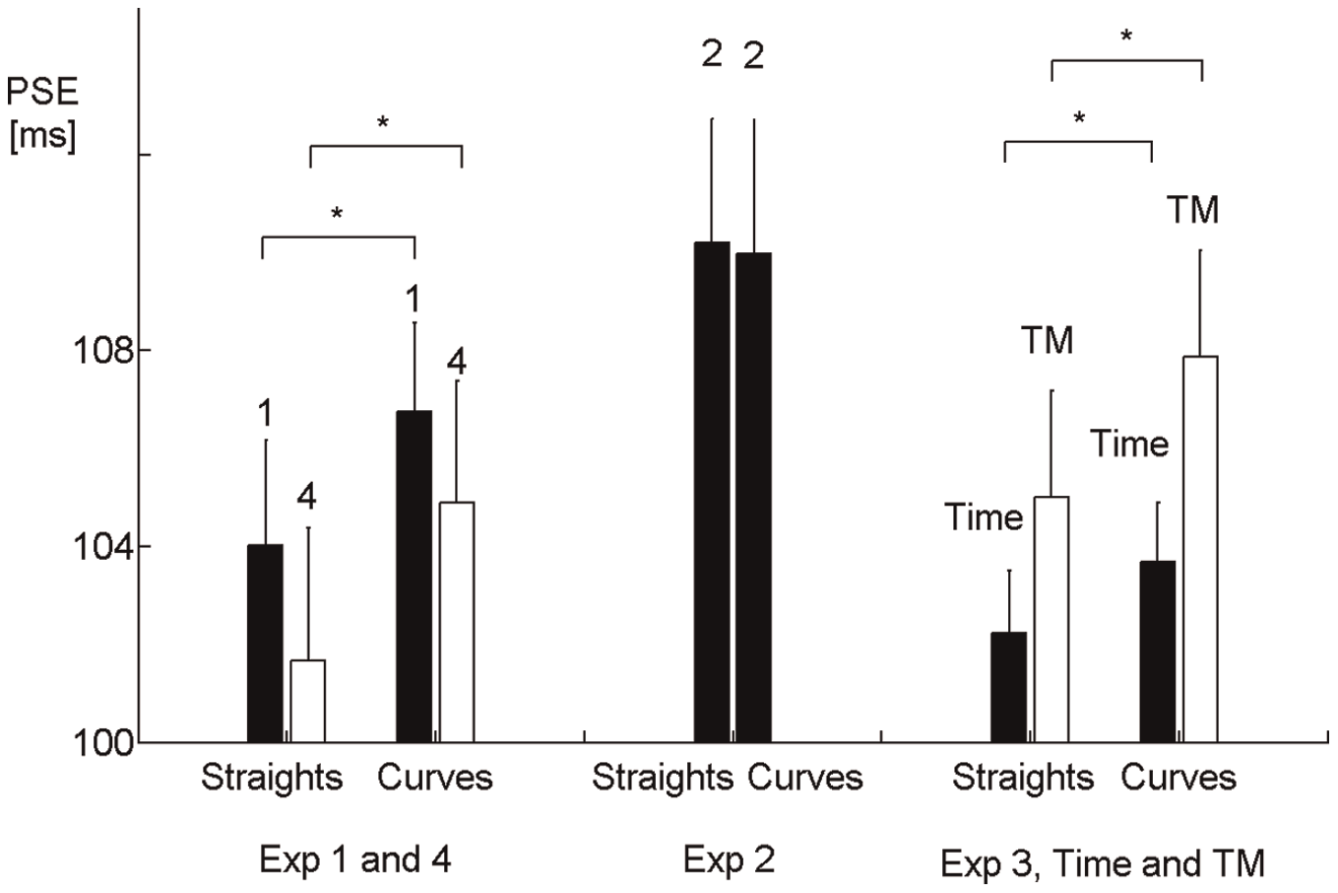
Distortion of subjective duration by perceived motion. Subjective duration (PSE) for the Straights and Curves condition, respectively, in Experiment 1 to 4. Error bars are standard errors, brackets with stars depict significant differences (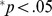). Both in Experiment 1 and 4 (left part) and in the Time and and Time-Motion condition (right part, Time-Motion abbreviated as “TM”), the PSE is significantly higher in Curves compared to Straights, indicating that durations were perceived as longer at the upper and lower straight compared to the left and right curve. In Experiment 2 (center part), there was no such effect.

First, we tested whether the visually guided arm motion extends an influence on duration discrimination of auditory intervals. Subjective duration may both be influenced by the speed and the curvature of the motion: If auditory representations of time are affected by the density of position changes, intervals presented during faster motion should be perceived as longer. On the other hand, if the density of control actions plays a critical role, intervals will be prolonged during more curved movements. To test both possibilities, we configured the target motion such that changes in curvature and speed of the motion were inversely related by a power law with an exponent of −1/3. This relation is naturally fulfilled in voluntary continuous motion [30]. Thus, the movement differed both in curvature and in tangential velocity during the presentation of *S*_1_ and *S*_2_. The PSE reflects the duration of the variable interval *S*_2_ at which it is perceived as equal to the constant interval *S*_1_. Thus, if the intervals in the Time-Motion condition are perceived as longer for faster motion, the PSE should be lower in Straights compared to Curves, as *S*_2_ is presented during faster motion in the straight apices. Conversely, if curvature prolongs subjective duration, the PSE should be increased in Straights compared to Curves. As shown in Figure 1, the PSE increased from Straights to Curves (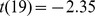, 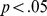, 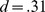). Additional analysis is reported in the section on Experiment 4, and in the Tables S1, S2, S3, S4. Thus, the hypothesis of an interaction between time perception and motor timing is supported. The direction of the distortion is consistent with motion speed being the distorting factor.

Furthermore, we tested whether participants actually followed the trajectory we prescribed by measuring curvature and velocity of the actual motion during the presentation of the *S*_2_ in the Time-Motion condition (see Supporting Text S1 for the details on these calculations). As expected, curvature increased (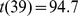, 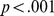, 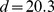) and velocity decreased (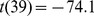, 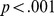, 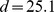) from Straights to Curves (see Table S5 and S6 for statistics on the motion data). The relation between curvature and tangential velocity could be well fitted to a power law with a mean exponent of 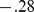 (SD .02), which is close to the prescribed −1/3. The PSE was correlated with both of those motion parameters (see Supporting Text S1).

### Experiment 2

**Figure 2:**
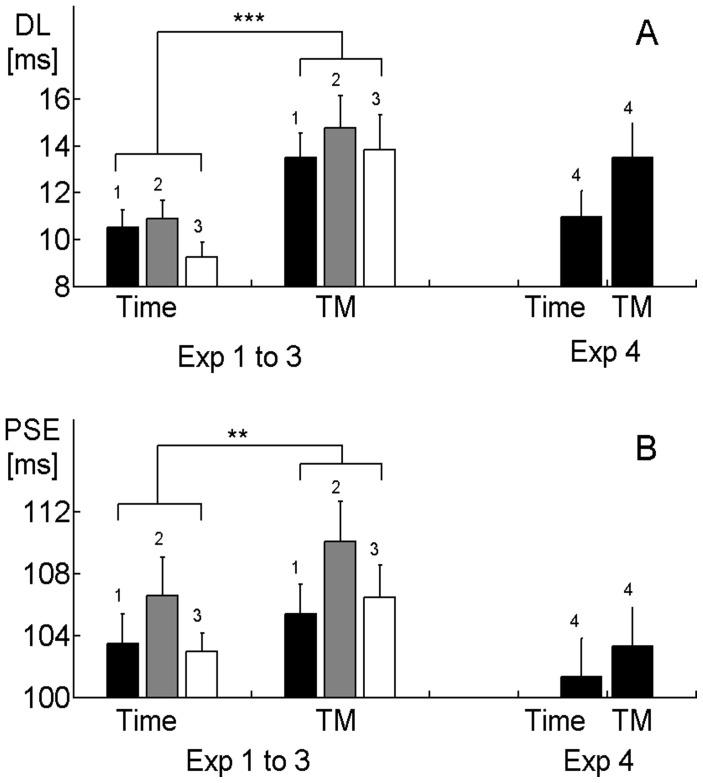
Dual task interference and training effects. Discrimination performance (DL, upper panel) and subjective duration (PSE, lower panel) compared between single-task (Time condition) and dual-task experiments (Time-Motion condition, abbreviated as “TM”) in Experiment 1 to 4. Error bars are standard errors, brackets with stars depict significant differences (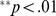, 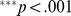). Both DL and PSE increase from single- to dual-task in Experiments 1 to 3 (left part), but not in Experiment 4 (right part).

From Experiment 1 alone, one can not determine whether the distortion in subjective duration is solely caused by the change of speed or of curvature. In Experiment 2, we disentangled these two possible causes by keeping the tangential velocity constant along the ellipse, so only curvature changed between conditions. If the distortion is caused by the changes of speed alone, it should be abolished by this manipulation. Indeed, the data from this experiment showed no PSE difference between Straights and Curves any more (Figure 1, 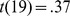, 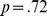). Analyzing the performed motion, however, we found that tangential velocity still decreased from Straights to Curves (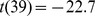, 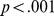, 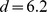) which is incompatible with the prescribed target motion. The relation between curvature and tangential velocity was again well fitted by a power law, although its exponent now deviated strongly from −1/3 (mean −.14, SD .06). This results are consistent with the finding that people tend to stick to the −1/3 power law relation [30], even when instructed otherwise [31], [32]. To compare Experiment 1 and 2 more directly, we performed a 2-way ANOVA with the within factor Condition (Straights vs. Curves) and the between factor Experiment (1 vs. 2). There was a significant effect of Experiment (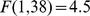, 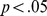, 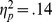), as the PSE is overall higher in Experiment 2 (Figure 2B). The factor Condition only showed a statistical trend (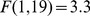, 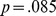, 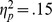). The was no interaction between the two factors (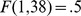, 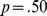).

As the PSE difference between Straights and Curves vanishes when there were no differences in target speed between conditions, we conclude that the distortion of subjective duration was caused by motion speed alone, with no influence of curvature. However, the speed of the performed motion still differed between conditions. This suggests that that the perceived motion may have induced the distortion, rather than the performed motion.

### Experiment 3

In visual time perception, it is known that moving stimuli are perceived as longer when the motion is faster [12], [14], [16]. Thus, auditory time perception could also be influenced by a cross-modal interaction of visual and auditory temporal representations, rather than by the active performance of the elliptic motion. This possibility is also consistent with the results of Experiment 2: While the arm movement of the participants changed in tangential velocity between Straights and Curves, the visual motion on the screen did not show such a change, at least for the target. Thus, one could interpret the missing distortion of the perceived duration in Experiment 2 as evidence that the visual motion affect the PSE more strongly compared to the performed motion. To test this explicitly, we performed Experiment 3, where we compared the distortion of subjective duration in the Time and the Time-Motion condition. To make these conditions truly comparable, we varied the *S*_2_ in the Time condition independently for the same four spatial configurations as in Time-Motion, based on the motion of the target instead of the resting proxy. That way, the same four conditions as in Time-Motion could be analyzed without any arm motion of the participants. There were 64 trials for each condition, resulting in a total number of 256 trials both in Time and Time-Motion.

The PSE increased from Straights to Curves both in the Time (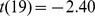, 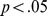, 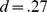) and the Time-Motion experiment (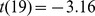, 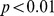, 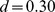) (Figure 1). To compare these two types of experiments more directly, we performed a 2-way ANOVA with the within factors Condition (Straights vs. Curves) and Experiment Type (Time vs. Time-Motion). There was a significant effect of Condition (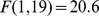, 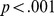, 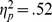), as the PSE distortion is present in both types of experiments (Figure 1, right panel). The factor Experiment Type showed a statistical trend (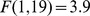, 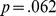, 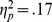), reflecting the somewhat lower PSE in Time compared to Time-Motion (Figure 1, right panel). Most importantly, there was no interaction between the two factors (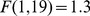, 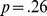). This lack of a difference of the distortion between Time and Time-Motion shows that a change in visual motion speed is sufficient to distort subjective duration, and that there is no additional effect from performing the motion.

### Experiment 4

The preceding experiments show that the distortion in subjective duration is caused by speed changes in perceived motion. A remaining question that was not addressed in former studies is whether the distortion may be due to attention rather than direct cross-modal interaction. The fact that our participants perform a secondary task allows us to directly test for this possibility: The mere presence of the motor task may constitute a source of interference which consumes attentional resources. Thus, if the distortion of subjective duration depends on attention, it should be affected by the level of cognitive load that the motor task extends. To that end, we first confirmed that the presence of the motor task induced dual-task inference, and then conducted an additional experiment where this interference is elevated by additional training of the motor task.

To demonstrate dual-task interference, we re-evaluated Experiment 1–3 by comparing the PSE and DL in the dual-task Time-Motion conditions (averaged over Straights and Curves) with the respective single-task Time conditions (Fig 2, see also tables S2 and S4). A secondary task performed concurrently with a time perception task is known to increase the variability and decrease the subjective duration of a time estimate [19], [20], an effect that is attributed to diminished attentional resources available for temporal processing. The increased variability should result in an increased DL in Time-Motion compared to Time experiment. The prolonged duration, however, is unlikely to increase the PSE values in our paradigm, as the perceived duration of both *S*_1_ and *S*_2_ would be affected. However, a possible way to use the PSE as a second measure of dual-task interference is opened by the observation that the PSE is consistently above the standard duration of 100 ms in all experiments (Fig. 1 and 2). This may be caused by the fact that in a series of two of more stimuli, the duration of first is typically overestimated [11], an effect that was related a decreased predictability of the first stimulus within a sequence [33]. Thus, diminished attention in the dual-task condition could lead to an overestimation of the *S*_1_, which is always presented at the first position. This would result in an decreased PSE in Time-Motion compared to Time.

Indeed, the comparison of the dual task and single task conditions in experiments 1–3 shows both a increase in the DL, Figure 2, 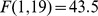, 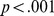, 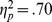) and a decrease in the PSE (Figure 2, 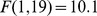, 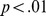, 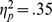). For the DL, these effects were also significant when experiments were analyzed individually, whereas the PSE only showed trends of differences for Experiment 2 and 3 (see tables S2 and S4). Thus, the dual-task interference is confirmed both by the DL and the PSE.

To explicitly test whether the distortion effect is affected by attention, we conducted Experiment 4 which includes an extended training phase for the motion task prior to the actual experiments. Participants practiced until they reached a defined level of tracking performance. Specifically, they had to keep the distance of the proxy from the target below a certain value over two minutes (see Supporting Text S1 for the details of the procedure). Such training has been shown to diminish dual-task interference on time perception [34], and it is assumed that the training leads to a more automatized performance of the secondary task, leaving more attentional resources for time perception. If the cross-modal distortion of subjective duration is independent from attentional resources, it should be unaffected by the training, while the dual-task effect will decline. In fact, neither the DL difference (Figure 2, 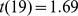, 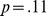) nor the PSE difference (Figure 2, 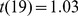, 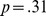) between single- and dual-task conditions was significant any more in Experiment 4. However, the PSE difference between Straights and Curves in the Time-Motion condition remained (Figure 1, 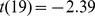, 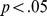, 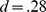). To make a more direct comparison between the situation with and without training, we performed a 2-way ANOVA with the within factor Condition (Straights vs. Curves) and the between factor Experiment (1 vs. 4) for the PSE. There was a significant effect of Condition (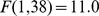, 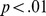, 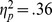), as both experiments showed the same PSE distortion (Figure 1), but there was no effect of Experiment (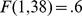, 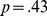) and also no interaction between the two factors (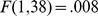, 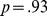).

Taken together, this analysis shows that the duration of subjective duration is not affected by attentional resources, because training diminished the dual task interference, but left the distortion between Straights and Curves unchanged.

## Discussion

In the present experiments, we consistently find that subjective duration of intervals presented in the auditory modality is influenced by a moving visual stimulus, in such a way that the perceived auditory duration is longer when the visual stimulus is moving faster. These results demonstrate a cross-modal interaction between visual and auditory temporal information. Our experimental paradigm combines an explicit auditory time perception task with visually guided motion. This allows us to observe cross-modal interactions that are more complex than the mere dominance of one modality over another: Because the visual modality contains no cues for the beginning and the end of the intervals to be timed, the estimate of time essentially depends on the auditory modality. But it also incorporates temporal information from vision, as demonstrated by a duration distortion that depends on the speed of the visually presented motion. As the effects of stimulus motion are well known in visual time perception [12], [14], we conclude that the change of the density of events induced by the change in visual motion speed carries over to the auditory domain. This is in contrast to the majority of studies on cross-modal duration cues, which suggest that auditory perception is dominant over visual perception in the time domain [2], [5], [6], [8]. Thus, although audition is known to be more precise for time perception compared to vision, the modality appropriateness hypothesis [21] does not seem to hold universally (see also [4], [7]).

Unlike former studies, we controlled for attentional factors by demerging the distortion effect from dual-task interference [19]. Thus, we conclude that duration distortion truly reflects cross-modal interaction and is not caused by the allocation of cognitive resources. The fact that we found dual-task interference for intervals in the range of 100 ms supports the emerging view that attentional factors affect temporal processing for intervals both above and below one second [28], challenging the notion of distinct mechanisms for time perception in these two domains [35], [36]. To our best knowledge, this is the first report of dual task interference in the milliseconds range induced by a motor task.

We did not find evidence that active performance of motion contributed to the speed-dependent duration distortion, which suggests a largely separated set of mechanisms for the timing of continuous motor acts and the perception of time. Former studies made the same proposal, but only reported the lack of correlations between perception and continuous motor timing [24]–[27]. Our results provide causal evidence for the notion that distinct “emergent” and “event” time representation govern continuous motor timing and both time perception and discrete motor acts, respectively [22]. This distinction should be further tested by applying a similar paradigm to time perception combined with discrete motion, where we expect a clear effect on subjective duration.

What are the implications of the present study for the nature of multi-sensory representations of time? At first glance, cross-modal interactions seem to support the classicial view that event time is represented by a centralized internal clock [13], [17], [18], which provides a unique representation of interval duration regardless of task or modality. However, this notion is discouraged by an increasing number of studies revealing highly modality-specific timing processes, residing e.g. in early vision [37]–[42]. The effect size of our results also argues against a centralized clock: In unimodal studies, visually presented motion at a speed comparable to our study distorted subjective duration by up to 400 ms [12], [14], even for intervals in the sub-second range [12]. If distortion acted directly onto a centralized clock, there would be no reason why the effect on auditory stimuli should be two orders of magnitude smaller. Ruling out a centralized timing mechanism does not imply the impossibility of amodal clocks, although we will have to consider how more detailed models of such timing structures could be constructed [4].

If one accepts that time is processed in a modality-specific way, a parsimonious explanation of cross-modal interaction can be formulated within the pacemaker-accumulator framework [13], [17], [18]. The model comprises a separate pacemaker in each modality [43], which emits pulses at frequencies that are modulated by the density of events in the respective modality [11], [12], [14], [15]. These pulses are then counted in a centralized “temporal hub”. The onset and offset of an interval to be represented trigger a switch which allows the accumulation of the pulses during the interval [44], so the duration of the interval can be estimated from the total number of pulses. A similar model has been proposed in the context of perceptual grouping [9]. Importantly, both the switch and the accumulating hub must be centralized. If each modality would generate a completely independent estimate of time [38], only modalities containing onset and offset information would be able contribute to the final estimate. This is incompatible with our observation that an ongoing flow of temporal information in the visual modality affects time perception in another modality, despite the absence of visual onset and offset cues.

A remaining question is how the temporal information from each modality is weighted. In its most extreme form, the modality appropriateness hypothesis would suggest that only the most reliable modality may contribute to the time estimate, while information from less reliable modalities is discarded. An alternative is given by Bayesian integration [1], which assigns the weights according to the relative reliability of each modality, and thus uses all available sources of information. It can be shown that this form of integration is optimal in terms of maximizing temporal information. Taking this information-theoretical view on time perception [2], [4], [45]–[47], the conflicting results regarding the dominance of one modality over another can be resolved: Detection of motion and dynamic changes is more reliable in vision compared to audition [48]. Consequently, studies involving dynamic manipulations observe dominance of vision over audition [4], [7], while static stimuli induce the opposite pattern [5]. Similarly, beneficial effects of congruent and distorting effects of incongruent rhythms [8] can be seen as the result averaging the temporal stimuli from both domains, which decreases variability when the information from both channels is the same [10], [45], but increases it when the information is conflicting. It remains to be shown, however, whether the Bayesian model can also quantitatively describe cross-modal integration [2], [4], [47].

In summary, our results demonstrate a direct interaction between visual and auditory time perception, while no connection between the timing of continuous motor acts and the perception of time can be found. The cross-modal interaction in the perceptual domain is consistent with Bayesian integration of temporal information from different sources into a temporal hub, according to the relative reliability of these sources. This kind of integration may be the brains’ solution to the problem of constructing unique and reliable representations of time despite of the fact that duration could be distorted by a large number of non-temporal stimulus features in each individual modality.

## Supporting Information

Figure S1 Setup of the experiment. Participants stood in front of a screen wearing headphones. They moved the end effector of a robotic manipulandum in order to move a proxy on the screen.
(EPS)

Figure S2 Sketch of the screen contents for visual feedback during the motor task. A blue sphere (target) moves along an elliptic trajectory at a prescribed speed. A red sphere (proxy) can be moved with a robotic manipulandum. Two ellipses surround the trajectory of the blue sphere to mark an area that should not be left by the red sphere. Numbers depict the four possible positions of the second auditory stimulus for the interval discrimination task. The first stimulus was presented at the previous apex. Position 1 and 3 are termed “Straights” throughout the paper, position 2 and 4 “Curves”. As an example, the black patches at Position 1 and 2 depict the case that the second stimulus is presented at the right curve. The arrows depict direction and magnitude of tangential velocity of the blue sphere in Experiment 1. The numbers, patches and arrows were not actually shown on the screen, and the ellipses were shown in yellow against a black background.
(EPS)

Table S1 PSE (in ms) for each individual condition and experiment. Each cell contains the average over all participants, and standard deviation in brackets. In Experiment 3, the Time and Time-Motion condition (abbreviated TM) are reported separately.
(PDF)

Table S2 PSE (in ms) for each experiment averaged for the Time, Time-Motion (abbreviated TM), Straights and Curves condition. Each cell contains the average over all participants, and standard deviation in brackets. In Experiment 3, the Time and Time-Motion condition are reported separately.
(PDF)

Table S3 DL (in ms) for each individual condition and experiment. Each cell contains the average over all participants, and standard deviation in brackets. In Experiment 3, the Time and Time-Motion condition (abbreviated TM) are reported separately.
(PDF)

Table S4 DL (in ms) for each experiment averaged for the Time, Time-Motion (abbreviated TM), Straights and Curves condition. Each cell contains the average over all participants, and standard deviation in brackets. In Experiment 3, the Time and Time-Motion condition are reported separately.
(PDF)

Table S5 Curvature for each experiment averaged for the Straights and Curves condition of the Motion and the Time-Motion (abbreviated TM) condition, respectively, measured in units of inverse screen units (one screen unit equals 30 cm on the computer screen). Each cell contains the average over all participants, and standard deviation in brackets.
(PDF)

Table S6 Velocity for each experiment averaged for the Straights and Curves condition of the Motion and the Time-Motion (abbreviated TM) condition, respectively, measured in units of screen units per second (one screen unit equals 30 cm on the computer screen). Each cell contains the average over all participants, and standard deviation in brackets.
(PDF)

Text S1 Information about data analysis, the training phase in Experiment 4, data on individual conditions, Experiment 1b, and correlations between time and motion data.
(PDF)
