# Supplementary material for: Cross-Modal Distortion of Time Perception: Demerging the Effects of Observed and Performed Motion
Source: PLoS One. 2012 Jun 12;7(6):e38092. doi: 10.1371/journal.pone.0038092 (PMC3373534; doi:10.1371/journal.pone.0038092)
Supplement: Table S1 — PSE (in ms) for each individual condition and experiment. Each cell contains the average over all participants, and standard deviation in brackets. In Experiment 3, the Time and Time-Motion condition (abbreviated TM) are reported separately. (PDF) [file pone.0038092.s003.pdf]

Exp	upper straight	right curve	lower straight	left curve
1	102.6 (10.1)	108.7 (8.2)	105.5 (9.7)	104.8 (8.3)
1b	105.1 (9.9)	108.6 (12.1)	101.1 (12.6)	107.7 (11.6)
2	110.8 (11.1)	110.1 (12.9)	109.7 (11.3)	109.7 (12.0)
3, Time	102.4 (6.2)	102.9 (6.1)	102.1 (5.5)	104.5 (5.0)
3, TM	105.2 (9.7)	109.5 (9.1)	104.9 (10.0)	106.3 (10.6)
4	100.1 (11.7)	105.7 (11.6)	103.2 (12.9)	104.1 (11.2)

Table S1. PSE (in ms) for each individual condition and experiment. Each cell contains the average over all participants, and standard deviation in brackets. In Experiment 3, the Time and Time-Motion condition (abbreviated TM) are reported separately.
