# Supplementary material for: Cross-Modal Distortion of Time Perception: Demerging the Effects of Observed and Performed Motion
Source: PLoS One. 2012 Jun 12;7(6):e38092. doi: 10.1371/journal.pone.0038092 (PMC3373534; doi:10.1371/journal.pone.0038092)
Supplement: Table S2 — PSE (in ms) for each experiment averaged for the Time, Time-Motion (abbreviated TM), Straights and Curves condition. Each cell contains the average over all participants, and standard deviation in brackets. In Experiment 3, the Time and Time-Motion condition are reported separately. (PDF) [file pone.0038092.s004.pdf]

Exp	TM	Time	Straights	Curves
1	104.0 (9.4)	105.4 (8.3)	103.5 (8.3)	106.8 (7.9)
1b	103.1 (10.4)	105.6 (10.8)	104.6 (8.0)	108.1 (11.6)
2	110.2 (11.0)	110.1 (11.3)	106.6 (10.6)	109.9 (11.8)
3, Time	102.2 (5.5)	106.4 (9.3)	103.0 (5.3)	103.7 (5.3)
3, TM	105.0 (9.4)	106.4 (9.3)	103.0 (5.3)	107.9 (9.5)
4	101.7 (11.9)	103.3 (11.0)	101.3 (10.8)	104.9 (10.8)

Table S2. PSE (in ms) for each experiment averaged for the Time, Time-Motion (abbreviated TM), Straights and Curves condition. Each cell contains the average over all participants, and standard deviation in brackets. In Experiment 3, the Time and Time-Motion condition are reported separately.
