# Supplementary material for: Cross-Modal Distortion of Time Perception: Demerging the Effects of Observed and Performed Motion
Source: PLoS One. 2012 Jun 12;7(6):e38092. doi: 10.1371/journal.pone.0038092 (PMC3373534; doi:10.1371/journal.pone.0038092)
Supplement: Table S4 — DL (in ms) for each experiment averaged for the Time, Time-Motion (abbreviated TM), Straights and Curves condition. Each cell contains the average over all participants, and standard deviation in brackets. In Experiment 3, the Time and Time-Motion condition are reported separately. (PDF) [file pone.0038092.s006.pdf]

Exp	TM	Time	Straights	Curves
1	14.2 (5.5)	13.5 (4.6)	10.5 (3.3)	12.8 (5.0)
1b	12.5 (3.6)	13.9 (4.4)	10.9 (4.4)	15.4 (5.6)
2	14.5 (6.4)	14.8 (6.0)	10.9 (3.4)	15.0 (6.4)
3, Time	8.8 (3.2)	13.8 (6.4)	9.3 (2.1)	9.7 (3.0)
3, TM	13.1 (5.9)	13.8 (6.4)	9.3 (2.8)	14.5 (8.2)
4	12.6 (5.6)	13.5 (6.4)	11.0 (4.8)	14.4 (7.9)

Table S4. DL (in ms) for each experiment averaged for the Time, Time-Motion (abbreviated TM), Straights and Curves condition. Each cell contains the average over all participants, and standard deviation in brackets. In Experiment 3, the Time and Time-Motion condition are reported separately.
