# Supplementary material for: Cross-Modal Distortion of Time Perception: Demerging the Effects of Observed and Performed Motion
Source: PLoS One. 2012 Jun 12;7(6):e38092. doi: 10.1371/journal.pone.0038092 (PMC3373534; doi:10.1371/journal.pone.0038092)
Supplement: Table S5 — Curvature for each experiment averaged for the Straights and Curves condition of the Motion and the Time-Motion (abbreviated TM) condition, respectively, measured in units of inverse screen units (one screen unit equals 30 cm on the computer screen). Each cell contains the average over all participants, and standard deviation in brackets. (PDF) [file pone.0038092.s007.pdf]

Exp	Motion, Straights	Motion, Curves	TM, Straights	TM, Curves
1	0.0157 (0.0009)	0.0022 (0.0002)	0.0158 (0.0008)	0.0022 (0.0001)
2	0.0131 (0.0010)	0.0029 (0.0007)	0.0133 (0.0017)	0.0027 (0.0004)
3	0.0154 (0.0007)	0.0022 (0.0002)	0.0156 (0.0012)	0.0023 (0.0002)
4	0.0158 (0.0006)	0.0042 (0.0042)	0.0161 (0.0009)	0.0023 (0.0001)

Table S5. Curvature for each experiment averaged for the Straights and Curves condition of the Motion and the Time-Motion (abbreviated TM) condition, respectively, measured in units of inverse screen units (one screen unit equals 30 cm on the computer screen). Each cell contains the average over all participants, and standard deviation in brackets.
