# Supplementary material for: Cross-Modal Distortion of Time Perception: Demerging the Effects of Observed and Performed Motion
Source: PLoS One. 2012 Jun 12;7(6):e38092. doi: 10.1371/journal.pone.0038092 (PMC3373534; doi:10.1371/journal.pone.0038092)
Supplement: Text S1 — Information about data analysis, the training phase in Experiment 4, data on individual conditions, Experiment 1b, and correlations between time and motion data. (PDF) [file pone.0038092.s009.pdf]

Supporting text S1

Data analysis. We used Microsoft Excel 2003 (Microsoft Cooperation) for collection and basic analysis of the data, MATLAB R2008b (The MathWorks) for calculation of the motion parameters and R 2.8.0 (The R Foundation for Statistical Computing) for statistical analysis of all the data. Effect sizes of statistical effects were computed by Cohen's d [1] for t tests and by partial η^2 [2] for analysis of variance, abbreviated as η_p^2 in the text.

Time task. The adaptive procedure used in the Time task was designed to let the duration of S_2 converge to the value where the participant judges the comparison interval as the longer one in 75% and 25% of all cases for S_2 starting above and below the standard interval S_1 duration of 100 ms, respectively. We calculated the mean S_2 for both of these runs in each condition (Time and the four positions in Time-Motion) based on the 20 last trials to exclude transient effects. From these values S_{75} and S_{25}, we calculated the difference limen (DL)

$$\mathrm{DL} = \frac{S_{75} - S_{25}}{2} \tag{1}$$

and the point of subjective equality (PSE)

$$\mathrm{PSE} = \frac{S_{75} + S_{25}}{2}. \tag{2}$$

Motion task. We analyzed the trajectories of the Motion task in two ways. First, we averaged over all points in the trajectory that correspond to a given angular position of the target. The target sphere took the finite number of 3142 such angular positions. For each of these angles, we calculated the mean and standard deviation of the deviation of the actual trajectory from the target trajectory Δr ("target deviation"), measured by the Euclidian distance

$$\Delta r = \sqrt{(x - \hat{x})^2 + (y - \hat{y})^2} \tag{3}$$

where x and y are the Cartesian coordinates of the proxy sphere and $\hat{x}$ and $\hat{y}$ are those of the target sphere.

The second way to analyze the trajectories was to cut them into pieces that correspond to individual revolutions, or laps. For constructing a lap, the angular position of the target sphere is not suitable, as the movement of the proxy is usually faster or slower than the target sphere, so no given set of 3142 subsequent data points could be expected to yield an exact lap. Instead, we determined the laps based to the variable radius of the ellipse. The first lap started at the first point where the radius was minimal with the y position greater than zero (upper apex), and ended at the next point where this condition was met again. For each data point of one of these laps, we calculated the curvature

$$\kappa = \frac{|x'y'' - y'x''|}{x'^2 + y'^2}^{3/2} \tag{4}$$

and the tangential velocity

$$\nu = \sqrt{\dot{x}^2 + \dot{y}^2} \tag{5}$$

where $\dot{x}$ and $\dot{y}$ are the approximated time derivatives of the x and y coordinate, respectively, and $x'$and y' are the approximated derivatives with respect to the angle θ around the origin of the ellipse, relative to the upper straight apex. In this way, the values of κ and ν are given as functions of the radius r of the trajectory of the proxy rather than the angular position of the target sphere. For each value of r, we average curvature and tangential velocity over all laps. Specifically, the values at the apices are determined by the four extreme values of the radius.

Training phase in Experiment 4 In Experiment 4, the training phase for the Motion task was prolonged such that all participants reached a common level of performance. We measured the target deviation Δr at each millisecond and updated a measure of the accumulated target deviation Δr_c in each time step t according to the following formula

$$\Delta r_c^{t+1} = \alpha \Delta r + (1 - 1/\tau)\Delta r_c^t \quad (6)$$

Δ_t started at an initial value of $\Delta_t^0 = 333$ cm (screen coordinates) and the training phase was run until Δr_c falls below half this initial value. Being updated by the above rule, Δr_c increases in each time step with the currently measured target deviation, weighted by an increase rate α (set to 2.5E-5 cm), and decays exponentially with a time constant τ (set to 2 min). At these values Δr_c decays to its half value (and stops the training phase) in 2 min if the participant keeps the target deviation at zero at all times. Non-zero target deviations increase Δr_c according to the weighting factor α. The value of results in a training phase that is not much longer than 2 min if the distance from the proxy to the target is kept below 7.5 cm at all times. That value was also used as a performance threshold in the Time-Motion condition (see above). Applying this procedure resulted in training phases with a mean length of 4.4 min (SD 2.3 min), as compared to 2.6 min (SD 0.9 min) in Experiment 1.

Data on individual conditions. In table S1 to S4, we present the mean and variance of the PSE and DL computed for each individual condition (table S1 for PSE and table S3 for DL), and the pooled values of PSE and DL over the two Straights and Curves conditions, respectively, and over all conditions within the Time and Time-Motion experiment, respectively (table S2 for PSE and table S4 for DL). We also included Experiment 1b (see below), and presented the Time and Time-Motion condition in Experiment 3 separately. Note that in some experiments, namely Experiment 1 and Experiment 3 (Time condition), the PSE is somewhat larger in the upper straight compared to the lower straight, violating the implicit assumption in our analysis of both Straights and Curves positions being identical, respectively. To resolve this possible issue, we performed Experiment 1b.

Experiment 1b. The PSE difference between upper and lower straight in some experiment may raise the concern that the observed PSE differences between Straights and Curves may be related to the absolute position of the target on the screen, rather than differences in visual motion speed as we concluded. To exclude this possibility, we performed a control experiment with 10 additional participants where we rotated the elliptic trajectory by 90 degrees such that the position of the target shifted for all conditions: The two Straights were now to the left and the right of the screen, and the Curves were positioned at the upper and lower part. If the position of either the visual target or of the arm movement during the auditory stimulus presentations affected subjective duration, the PSE would change in opposite way in this setup compared to the original experiment, or at least show a less pronounced distortion. In contrast to this hypothesis, the PSE increased from Straights to Curves, just as in Experiment 1 ($t(19) = -3.1$, $P < 0.006$, Cohen's $d = 0.46$). From this result, we conclude that absolute spatial position has no notable effect on the PSE compared to visual motion speed.

Correlations between time and motion data. To directly correlate time and motion data, we extracted those pieces from the trajectories which were passed during the presentation of the two auditory stimuli of each trial in Experiment 1. We calculated the average of curvature and tangential velocity for the trajectory piece corresponding to the first and the second stimulus, respectively. Then, we correlated the difference in these measures between to first and second tone with the PSE and the DL. The PSE was significantly correlated with the differences of both parameters of motion between the first and the second stimulus (curvature: $r = -0.33$, $t(68) = 3.1$, $P < 0.003$, tangential velocity: $r = 0.32$, $t(68) = 3.0$ $P < 0.004$), but there was no significant correlation of the DL with neither the curvature ($r = 0.16$, $t(68) = 1.4$, $P = 0.16$) nor the velocity differences ($r = -0.16$, $t(68) = 1.5$, $P = 0.15$).

References

1. Cohen J (1988) Statistical power analysis for the behavioral sciences (2nd edition). New York: Academic Press.

2. Keppel G (1991) Design and analysis: A researcher's handbook. Englewood Cliffs, NJ: Prentice Hall.
